# Elastosis in ERα-positive male breast cancer

**DOI:** 10.1007/s00428-020-02920-7

**Published:** 2020-09-15

**Authors:** Marijn A. Vermeulen, Carolien H. M. van Deurzen, A. Elise van Leeuwen-Stok, Paul J. van Diest

**Affiliations:** 1 Department of Pathology, University Medical Center Utrecht, PO Box 85500, 3508GA Utrecht, The Netherlands; 2 Department of Pathology, Erasmus MC Cancer Institute, Erasmus University Medical Center, Rotterdam, The Netherlands; 3 BOOG Study Center/Dutch Breast Cancer Research Group, Amsterdam, The Netherlands

**Keywords:** Breast cancer, Male, Elastosis, Estrogen receptor

## Abstract

In female breast cancer (BC), elastosis is strongly related to estrogen receptor alpha (ERα) expression. Male breast cancers almost invariably express ERα; so, the aim of this study was to investigate elastosis frequency in invasive male BC as well as clinicopathological correlations, in comparison with females. A total of 177 male BC cases and 135 female BC cases were included, all ERα-positive and invasive carcinoma of no special type. Elastosis on H&E-stained slides was scored in a four-tiered system as elastosis grade (EG) 0 (no elastosis) to EG3 (high amount of elastosis). EG scores in male BC were correlated to histopathological characteristics and overall surviva and compared with female BC EG scores. Male BC showed some degree of elastosis in 26/117 cases (22.2%) with none showing EG3, while female BC cases showed elastosis in 89/135 cases (65.9%) with 21.5% showing EG3 (*p* < 0.001). This difference retained its significance in multivariate logistic regression. In male BC cases, no significant correlations were found between the amount of elastosis and age, grade, mitotic activity index, and PgR. In addition, no significant prognostic value of elastosis was seen. In conclusion, despite high ERα expression, male BC showed significantly less elastosis than female BC. Elastosis did not show clinicopathological correlations or prognostic value. Therefore, elastosis seems to be a less useful ERα tissue biomarker with less clinical significance in male BC compared with females, pointing towards important BC sex differences.

## Introduction

Elastic fibers are composed of two important components: elastin and small microfibrils. The precursor tropoelastin is secreted by fibroblasts, chondrocytes and smooth muscle cells, and this protein is crosslinked by one of the lysyl oxidase family members. The microfibrils are thought to function as a scaffold to facilitate this. Cross-linked aggregates form larger structures and eventually form a functional elastic fiber, providing elastic recoil to several different tissues [1]. Large aggregates of these elastic fibers in breast cancer (BC) are called elastosis.

Elastosis is a well-known phenomenon in female BC and has been studied for decades. The biological background of elastosis in the breast is not well understood but it is suggested that the elastic fibers are not produced by only fibroblasts, but also by endothelial cells and neoplastic epithelial cells [2]. It can be observed in the periductal and perivascular spaces or diffusely in the tumor stroma. Shivas and Douglas categorized elastosis in 1972 into four grades with grade 0 corresponding to no elastosis and grade 3 corresponding to numerous dense aggregates of elastic fibers and found a favorable survival in female breast cancers showing a high amount of elastosis [3]. This correlation of elastosis with survival or favorable tumor characteristics such as low grade and Ki67 index has later been confirmed by different groups, although other groups could not confirm this [4–6]. Another well-known correlation is that of elastosis with expression of the estrogen receptor alpha (ERα). In ERα-positive tumors, a high amount of elastosis can be found, compared with ERα-negative tumors that show less elastosis [4, 5, 7].

An estimated 2670 men will develop BC in the USA in 2019, which is almost 1% of the total number of estimated new breast cancer cases, making male BC a rare disease [8]. Previous studies have shown similarities, but certainly also differences between BC in males compared with females. For instance, there is a difference in distribution of histologic as well as molecular subtypes; men tend to present with BC at a higher age and present with more advanced disease at presentation compared with women [9–12]. In addition, important differences at the molecular and epigenetic level have been described [13, 14].

BC in males is almost invariably ERα positive, but because of the important differences between male and female BC, it cannot just be assumed that elastosis in male BC occurs in a similar frequency and show the same clinicopathologic correlations as in female BC. In the present study, our aim was therefore to establish the frequency of elastosis in ERα-positive male BC and to correlate the degree of elastosis to clinicopathological features and prognosis in comparison with ERα-positive female BC cases.

## Materials and methods

### Patient material

Male patients with ERα-positive invasive BC were selected from the Dutch part of the EORTC 10085/TBCRC/BIG/NABCG International Male Breast Cancer Program [15, 16], which was conducted as global effort to retrospectively assess tumor tissue of men diagnosed with breast cancer between 1989 and 2009. Male patients in The Netherlands were identified through the Dutch Cancer Registry. Paraffin-embedded male BC tissue was retrospectively collected by the Dutch Breast Cancer Research Group (BOOG). Archival tissue of all patients was handled according to the Dutch Code for Proper Use of Human Tissue (www.federa.org). A subgroup of this initial population was selected based on at least one available hematoxylin and eosin (H&E)-stained slide and known ERα status. All patients were diagnosed with invasive carcinoma (IC) of no special type (NST, according to the 2012 WHO), resulting in 117 male patients, all with one available H&E-stained slide containing tumor [17]. To match with this, one representative H&E-stained slide was selected from all 135 female patients with ERα-positive IC NST collected between 2017 and 2018 at the Department of Pathology of the University Medical Center Utrecht, Utrecht, The Netherlands. One slide of each tumor was chosen that showed the highest tumor content of all H&E-stained slides. Elastosis degree was not taken into account when choosing the slide. All H&E-stained slides contained a full cross section of the tumor.

Patient and tumor characteristics including age at diagnosis were recorded and the H&E-stained slides were reviewed by two experienced pathologists to confirm the diagnosis and to assess the degree of elastosis. Consensus was reached in all cases. Unfortunately, sufficient data on lymph node status and presence of lymphovascular invasion was not available for the male BC cases; so, these factors could not be taken into account. The pT stage was known for most male BC cases. The pT stage was based on the TNM 8 classification [18]. The tumors were graded according to the modified Bloom and Richardson score [19]. ERα, PgR, and HER2 were evaluated using immunohistochemistry and scored according to ASCO-CAP guidelines [20]. ERα and PgR were considered positive when > 10% of the tumor cells showed positive staining. Survival data was available for male BC cases but not for female BC cases. Survival outcome was defined as death due to any cause. The average length of follow-up was 8.32 years.

### Quantification of elastosis

**Fig. 1:**
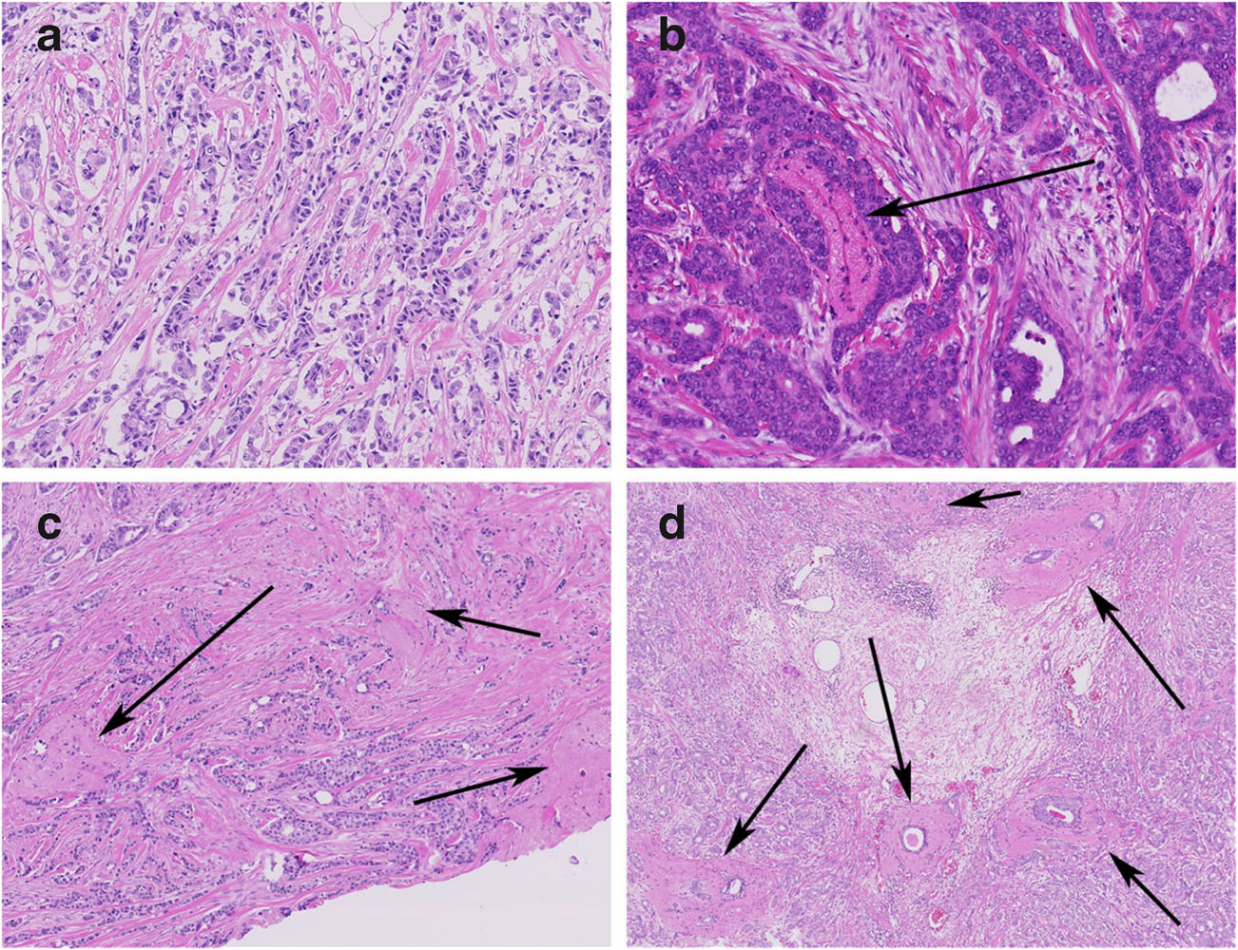
Elastosis in invasive male breast cancer, which can be identified in the H&E staining and classified using a four-tiered system. Elastosis grade 0: no elastosis can be seen (**a**), elastosis grade 1: only one small field of elastosis was found in this tumor (**b**), elastosis grade 2: 5 fields of elastosis were found in this tumor, of which 3 are shown in this image (**c**) and elastosis grade 3: this tumor demonstrated a high amount of elastosis with a big confluent field of elastosis shown here (**d**)

Elastosis was assessed in a process resembling usual diagnostics: digital (scanned) slides were screened at a magnification of 5×, and areas suspected for elastosis were additionally assessed at 10–20× for confirmation. Elastosis was quantified using a four-tiered system, according to the degree of elastosis observed on the H&E-stained slide. This system was based on the system described by Shivas and Douglas in 1972 on elastica stains where Elastica Index 0 correlated to a total absence of elastosis, Elastica Index + to an occasional clump of tumor cells invested by a fine mantle of elastica, Elastica Index ++ to more numerous groups of tumor cells with a think surrounding zone of elastica, and Elastica Index +++ to numerous thick and dense aggregates of elastica [3]. We modified this system to fit to H&E stains as we did not have elastica stains or tissue blocks available, but only H&Es. One representative slide per patient was examined as described above. Elastosis can be seen as clumps of elastic fibers that appear as an acellular area, usually surrounding ducts or tumor fields, in H&E-stained sections. This is easily distinguished by experienced pathologists from fibrosis or desmoplastic stroma, as elastosis appears as an eosinophilic to grayish area (like elastosis solaris in the skin) and appears as a well circumscribed area, and not as diffuse changes in the stroma. The areas of elastosis varied in size from approximately 0.3 to 1.2 mm, although size was not a criterion that was used. Elastosis grade (EG) 0 corresponded to no demonstrable elastosis, EG1 corresponded to 1 to 3 single ducts or groups of tumor cells surrounded by elastosis, EG2 to 4–6 single ducts or tumor cells surrounded by elastosis, or 2–3 bigger and confluent fields of elastosis, and EG3 corresponded to > 6 single ducts or groups of tumor cells surrounded by elastosis or > 3 confluent fields of elastosis (Fig. 1). To validate our morphological scoring of elastosis in H&E sections, 19 female BC cases with varying degree of elastosis on H&E were stained with an Elastica von Gieson stain: 5 cases with EG0, 4 with EG1, 5 with EG2, and 5 with EG3.

### Statistics

Statistical calculations were performed using SPSS for Windows version 25. *P* values of < 0.05 were regarded as significant. For correlations between categorical variables, Pearson *χ*^2^ test (or Fisher’s exact test when appropriate) was used. Continuous variables were analyzed using the *t* test. Multivariate analysis was done with logistic regression, taking the clinicopathological features that showed significance with univariate analysis into account. Survival analysis was done by plotting a Kaplan-Meier survival curve and assessing significance with logrank test. Multivariate survival analysis was done with Cox regression.

## Results

### Clinicopathological features

All patients, male (*n* = 117) and female (*n* = 135), had invasive BC of no special type (NST) and all tumors were ERα positive. Histopathological features of the male BC cases and female BC cases are summarized in Table 1. The male patients had a median age of 65.4 years (28–98 years), compared with a median age of 58 years (35–79 years) for females (*p* < 0.001). Male BC was more frequently graded as a histologic grade 2 (55.6%) compared with female BC (34.1%), which showed a higher percentage of grade 1 and grade 3 cases (*p* = 0.008 for grade 1 versus grade 2, *p* = 0.02 for grade 2 versus grade 3, *p* = 0.600 for grade 1 versus grade 3). The Mitotic Activity Index (MAI) was also significantly different with a lower mean MAI in male BC compared with female BC (7.45 versus 10.13, respectively, *p* = 0.011). The pT stage was significantly different between males and females (*p* < 0.001). In subanalysis, this difference is mainly between pT1 and pT2 (*p* = 0.002), pT1 and pT4 (*p* < 0.001), pT2 and pT4 (*p* < 0.001), and pT3 and pT4 (*p* = 0.002). PgR was positive in significantly more male BC cases compared with female BC cases (*p* ≤ 0.001).

**Table 1:** Clinicopathological features of male and female breast cancer patients. Missing data were excluded in the given percentages

Feature		Male (*n* = 117)	Female (*n* = 135)	*p* value
Age	Mean	64.4	58.8	*< 0.001*
Grade	I	31 (26.5%)	49 (36.3%)	*0.02*
	II	65 (55.6%)	46 (34.1%)	
	III	21 (17.9%)	40 (29.6%)	
pT stage	1	51 (45%)	100 (75%)	*< 0.001*
	2	100 (35%)	31 (23%)	
	3	1 (1%)	3 (2%)	
	4	21 (19%)	0 (0%)	
	*Missing*	5	1	
Mitoses/2mm2	Mean	7.45	10.13	*0.011*
	0–8	85 (72.6%)	70 (51.9%)	*0.001*
	> 8	32 (27.4%)	65 (48.1%)	
PgR	neg	4 (3.4%)	29 (21.5%)	*< 0.001*
	pos	112 (96.6%)	106 (78.5%)	
	*Missing*	1	0	
HER2	neg	100 (89.3%)	127 (94.1%)	0.241
	pos	12 (10.7%)	8 (5.9%)	
	*Missing*	5	0	
Elastosis	0	91 (77.8%)	46 (34.1%)	*< 0.001*
	1	20 (17.1%)	32 (23.7%)	
	2	6 (5.1%)	28 (20.7%)	
	3	0	29 (21.5%)	
Elastosis (2 categories)	0 + 1	111 (94.9%)	78 (57.8%)	*< 0.001*
	2 + 3	6 (5.1%)	57 (42.2%)	

### Elastosis in male versus female breast cancer

**Fig. 2:**
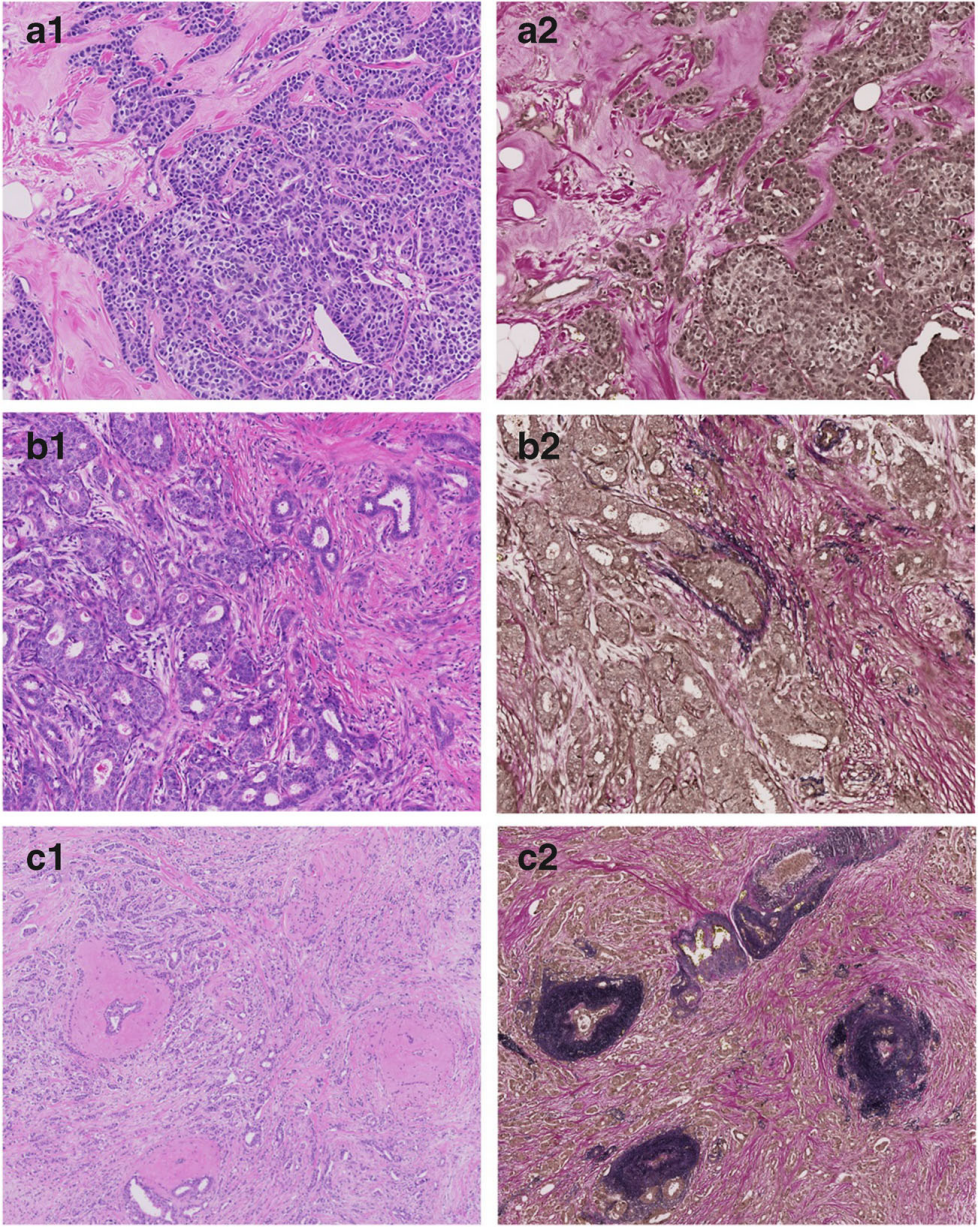
Validation of elastosis scoring in H&E (1) stained sections by parallel elastic staining (2) in a subset of three female breast cancer cases. **a** An EG0 case (on both H&E and Elastica von Gieson (EVG) stain). **b** EG0 on H&E and EG1 on the EVG stain with a small rim of elastin. **c** An EG3 case (on both H&E and EVG stain)

The elastosis scores for the 19 female BC cases that were stained with an Elastica von Gieson stain showed good correlation to elastosis scoring on the H&E slide, validating our H&E scoring system. In 15/19 cases, we had a perfect match between elastin stain scoring and H&E scoring of elastosis, 3 cases were scored negative on H&E (EG0) but showed a tiny rim of elastosis around one duct (EG1), and 1 case was scored EG2 on H&E but was found to have more elastosis on the elastin stain (EG3). Figure 2 illustrates this validation; Table 2 shows the data.

**Table 2:** Validation of elastosis scoring on Hematoxylin&Eosin (H&E) staining by elastic Von Gieson (EVG) staining

Case	Elastosis grade on H&E	Elastosis grade on EVG	Concordant (yes/no)
1	0	1	No
2	0	0	Yes
3	0	1	No
4	0	0	Yes
5	0	1	No
6	1	1	yes
7	1	1	Yes
8	1	1	Yes
9	1	1	Yes
10	2	2	Yes
11	2	2	Yes
12	2	3	No
13	2	2	Yes
14	2	2	Yes
15	3	3	Yes
16	3	3	Yes
17	3	3	Yes
18	3	3	Yes
19	3	3	Yes
Concordance			15/19 (79%)

Table 1 shows EG scores in male and female BC. Using the four-tiered system of grading elastosis, a significant difference was found between male and female BC. Male BC showed in general a lower amount of elastosis (*p* < 0.001). Male BC showed at least some degree of elastosis in 26/117 cases (22.2%) with no cases showing EG3, while female BC cases showed elastosis in 89/135 cases (65.9%) with 21.5% showing EG3 (*p* < 0.001). When comparing EG0/1 to EG2/3, significance remained (*p* < 0.001). This difference between male and female BC was found in subgroups of histologic grade 1 and grade 2 tumors (*p* < 0.001 for both), but not in grade 3 tumors (*p* = 0.199).

In logistic regression considering age, MAI, grade, PgR, and elastosis (EG0/1 versus EG2/3), elastosis was the highest predictor for gender (*p* < 0.001, HR 22.487, 95% CI 8.319–60.782).

### Elastosis in male breast cancer: correlation with histopathological features and overall survival

**Fig. 3:**
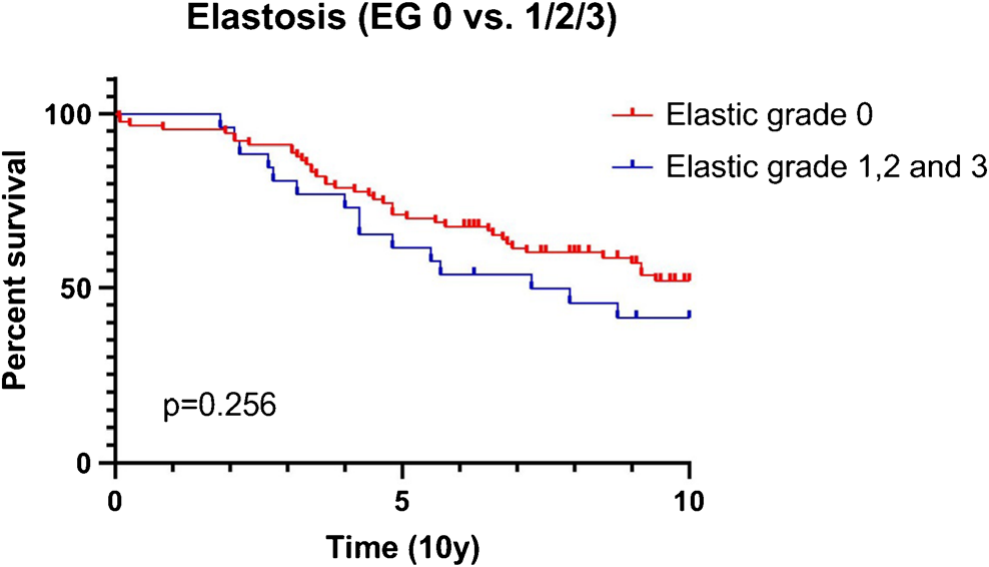
Kaplan-Meier survival curves of invasive male breast cancer according to the amount of elastosis

No significant differences were found between the amount of elastosis and grade (*p* = 0.651), pT stage (*p* = 0.331), age (cut-off 55 years, *p* = 0.276) MAI (cut-off 8, *p* = 0.613), PgR (*p* = 0.834), or HER2 (*p* = 0.668). In univariate analysis and multivariate analysis, there was no significant difference in 10-year survival for any elastosis (EG1/2) versus no elastosis (EG0) in male BC. The Kaplan-Meier survival curve is shown in Fig. 3.

## Discussion

Breast cancer is a well-known and well-studied disease as it is the leading type of cancer in women worldwide, accounting for approximately 30% of the estimated new cases of cancer in the USA in 2019 [8]. In contrast to female BC, male BC is understudied and unfamiliar among the public due to its low prevalence. Of all the breast cancers diagnosed in the USA in 2019, only 1% occurs in the male breast [8]. When male BC is diagnosed, it is usually treated using treatment algorithms derived from female BC studies. Male BC, however, is not as similar to female BC as one might assume, as previous studies have shown differences in the distribution of histologic as well as molecular subtype, age at presentation, and differences at the molecular and epigenetic level [9–14].

In female BC, it is well shown that the presence of elastosis is correlated to ERα expression [4, 5, 7]. To the best of our knowledge, this correlation had not been studied in male BC before. We therefore studied elastosis in 117 male BC cases, validated H&E scoring by Elastica von Gieson staining in a subset of female cases, and correlated the amount of elastosis to histopathological characteristics and survival. In addition, we compared our results to 135 female BC cases. All cases were ERα positive and all cases were subtyped as invasive carcinoma of no special type, according to the WHO. The pT stage differed significantly between male and female breast cancer, mainly caused by the high number of pT4 cases in males (19%) compared with females (0%) and, as a result, a lower number of pT1 cases in males (45%) compared with females (75%). As pT4 includes cases with skin ulceration and/or chest wall invasion and these symptoms are described to be relatively frequent in males, this is a likely explanation for this finding [21].

In our study, none of the male BC cases showed an abundant amount of elastosis (EG3). Only six cases (5.1%) showed a moderate amount of elastosis (EG2). This was significantly lower than the number of female BC cases showing EG2 and EG3 (20.7% EG2 and 21.5% EG3). A previous female BC study examining elastosis using a similar grading scheme (grades 0, 1, 2, and 3) found 16.5% of the 272 cases to show a high amount of elastosis (EG3). A total of 33.8% showed no elastosis (EG0), 28.7% a minimal amount (EG1), and 21.0% a moderate amount (EG2) [4]. This distribution is similar to our female BC study population and strengthens our finding that the stroma in male and female BC differs, even in multivariate analysis. A limitation of this study is that we could only examine one H&E-stained slide of the male BC cases, and some degree of heterogeneity in the tumor cannot be excluded. To minimize the possible bias due to this issue, we also scored only one slide in the female BC cases for fair comparisons.

In breast cancer, the production of the elastic fibers is thought to originate from both neoplastic epithelial cells as well as from (myo-)fibroblasts [2, 3, 22, 23]. A previous study using in situ hybridization for elastin mRNA on BC sections and using BC cell lines to examine elastin biosynthesis and regulation in fibroblasts and epithelial cells showed that the regulatory mechanism of elastin biosynthesis is probably similar to the mechanism in normal elastotic fibroblasts. Cells that showed to produce immunoreactive tropoelastin were epithelial cells, fibroblasts, and endothelial cells, with usually more than one cell type involved per studied sample [2]. As the immunoreactive epithelial cells were located at the periphery and in close proximity to stroma, it is believed that the interaction between the stroma and epithelial cells triggers tropoelastin biosynthesis in the epithelial cells [2]. Other studies have also shown the importance of the stroma/extracellular matrix (ECM) in breast cancer [24, 25]. That elastosis is common in ERα-positive female BC is a well-known fact, but the underlying mechanism of this correlation has not been described to our knowledge. As ERα is known to have influence on gene expression including many genes, perhaps one could speculate that in men, certain genes that play a role in elastic fiber formation are expressed differently compared with women or are more susceptible to ER influence, resulting in lower elastic fiber formation, and as a consequence, a lower amount of elastosis [26]. Why the association between ERα and elastosis is different in male BC remains an unanswered question and further research is needed. This could be investigated by looking at stromal gene signature, which has been done in a previous study revealing different signatures for different stromal elements [27].

In addition to our comparison of elastosis in male and female BC, we correlated the amount of elastosis to different histopathological features, but no significant correlations were found between elastosis and histologic grade, pT stage, age, MAI, and PgR. The relationship between tumor size and stromal elastosis has been analyzed in a previous study by Chen et al. that found no significant correlation in female BC [4].

Also, no significant prognostic value of elastosis was seen, although a limitation of this study is that we could only use overall survival. Breast cancer-specific survival was not available. In female BC, the correlation between survival and elastosis differs, as several studies found an improved survival in cases with a high amount of elastosis but others found no correlation or an inversed one [3–6].

In conclusion, despite high ERα expression, male BC shows significantly less elastosis than female BC with no relevant clinicopathologic correlations or prognostic value. Therefore, elastosis seems to be a less useful ERα tissue biomarker with less clinical significance in male BC than in female BC, again pointing towards important BC sex differences. Although male BC is a rare disease, further research is needed to better understand the underlying pathogenesis of (lack of) elastosis in male BC.
